# Laparoscopic Hepatectomy via Remote Mentoring From Jamaica to Trinidad

**DOI:** 10.7759/cureus.20177

**Published:** 2021-12-05

**Authors:** Shamir O Cawich, Lindberg Simpson, Andrew Josephs

**Affiliations:** 1 Surgery, University of the West Indies, St. Augustine, TTO; 2 Surgery, Kingston Public Hospital, Kingston, JAM

**Keywords:** partial hepatectomy, laparoscopic hepatectomy, liver parenchymal transection, liver surgeon, advanced laparoscopy

## Abstract

Laparoscopic hepatectomy brings many physiologic advantages over open hepatectomy and adheres to all oncologic principles. It is currently considered the standard of care. However, these are technically difficult operations to perform. Consequently, the expertise may not be universally available for all patients to benefit from laparoscopic hepatectomy. We report a unique situation where remote mentoring was used to guide bariatric surgeons in Jamaica to complete a laparoscopic hepatectomy.

## Introduction

Although laparoscopic hepatectomy is now widely accepted as standard of care [1], it is a complex operation that requires advanced skill sets to perform. These advanced laparoscopic skill sets are not universally available in order for patients who require hepatectomy to benefit from the laparoscopic approach [2]. This is the scenario in many countries in the English-speaking Caribbean [3]. 

We encountered a unique situation where laparoscopic bariatric surgeons in Jamaica, with no experience in hepatectomies, performed an emergency non-anatomic left hepatectomy via remote mentoring. When cases are carefully selected, remote mentoring may be a way to promote minimally invasive procedures in countries where human resources and expertise are limited [3].

## Case presentation

**Figure 1:**
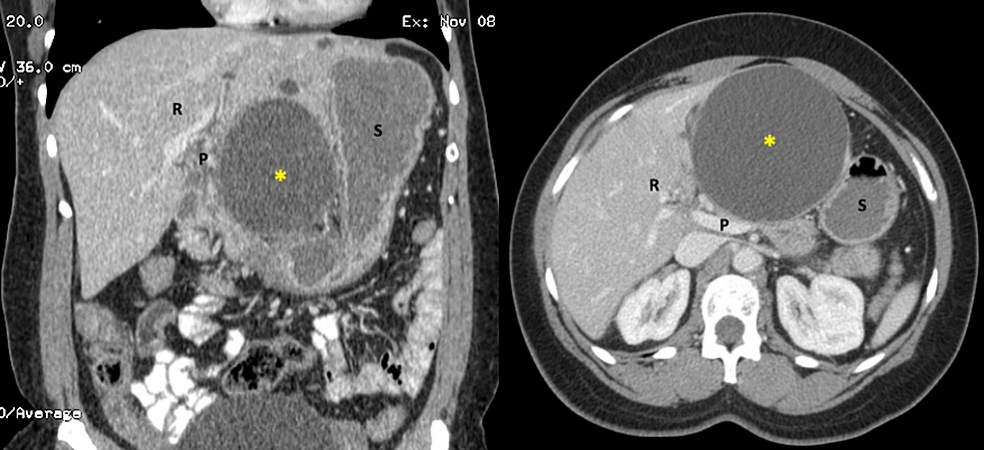
CT scan images revealing a large, ruptured left hepatic cyst. The large 12x15 cm ruptured hepatic cyst (asterix) is visible. The stomach (S) is draped over the cyst to contain the rupture along with the right liver (R).

A 45-year-old woman presented to an emergency room in Jamaica complaining of sudden upper abdominal pain. She was stabilized and investigated with CT scan that revealed a complex giant left hepatic cyst, with mesenteric stranding and free fluid suggesting contained rupture (Figure 1).

This patient required emergency non-anatomic left hepatectomy, but a hepatobiliary surgeon was not immediately available. The attending bariatric surgeon contacted a hepatobiliary surgeon in Trinidad who offered to assist via remote mentoring.

**Figure 2:**
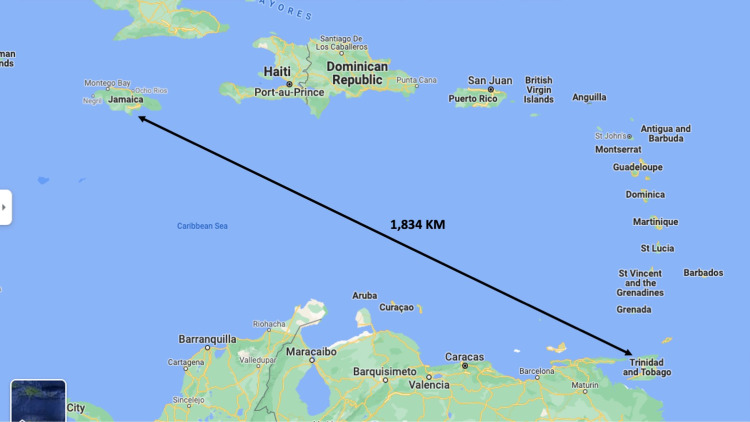
Google Satellite Maps® showing spatial orientation between the two countries involved in the remote-mentoring exercise. The remote mentor in Trinidad guided the operating surgeon 1834 km away in Jamaica.

The hepatobiliary surgeon organized dedicated time to be virtually present during the entire operation. He used computers equipped with FaceTime software (Apple Inc., Cupertino, CA, USA) to communicate in real time from 1,834 km away in Trinidad (Figure 2).

**Figure 3:**
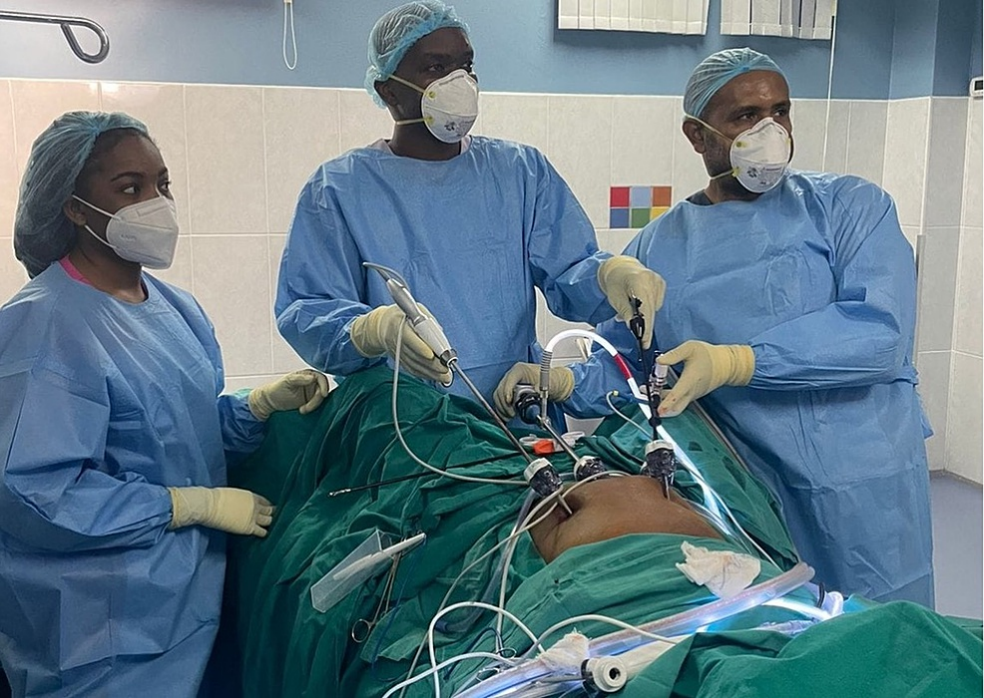
Photo documentation of the OR arrangement during remote mentoring. A laparoscopic stack was parked at the head of the operating table in a position for the primary surgeon to have an unobstructed view. One smartphone camera captures a view of the surgical field. OR, operating room.

In Jamaica, the primary surgeon stood between the patient's legs, comfortably able to visualize the working monitors (Figure 3). A previously described technique utilizing Apple iPhones® [4] was used to stream live feed of the laparoscopic video and the operating field to the remote mentor.

**Figure 4:**
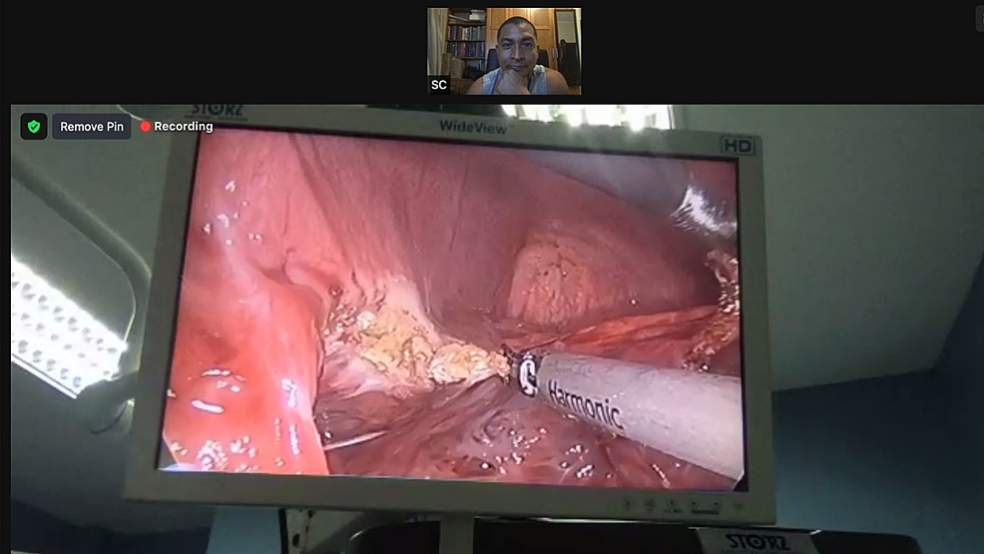
A real-time view of the laparoscopic feed as viewed from the remote mentor’s screen as he observes the parenchymal transection.

The remote mentor virtually observed the operating field and gave instructions to choose resection margins and transect hepatic parenchyma using ultrasonic dissectors [4] (Figure 4).

**Figure 5:**
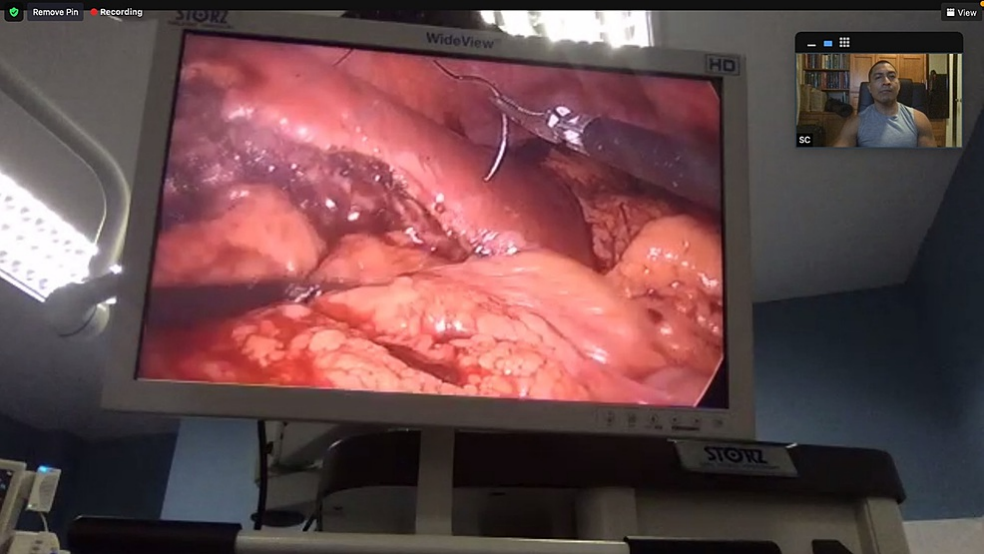
A real-time view of the operating field as seen by the remote mentor. The operating surgeon has completed resection and now sutures omentum to the parenchymal transection line.

The non-anatomic laparoscopic hepatectomy was completed uneventfully. An apron of omentum was sutured to the parenchymal transection line (Figure 5) and drains left in situ. After the operation was completed, the patient spent 24 hours in the ICU and a further five days in the surgical wards. She was sent home on post-operative day 6 after an uneventful period of recovery. 

## Discussion

The scenario described in this case report is common in developing countries, where sub-specialty expertise may not be immediately available for laparoscopic hepatectomies. Some patients may opt to travel to developed countries, but many do not have this opportunity due to financial barriers [5]. We propose that distance mentoring may be a solution when there are competent surgeons who possess the necessary advanced skill sets to complete laparoscopic hepatectomies. Mentoring is the well-accepted concept where an experienced surgeon coaches a less experienced colleague [4-6]. We extended the traditional mentoring concept to one where the mentor only present virtually [4].

The surgeons participating in this exercise had previously operated together physically and were familiar with each others' personalities, judgment, and technical abilities. It is important for all parties to be familiar with each other in order to determine whether the operating surgeon would be able to manage a potential complication without the mentor being physically present [3,4].

For a successful exercise, the remote mentor must be (1) well experienced, (2) well trained, (3) patient, and (4) have emotional intelligence [3,4]. Since technical maneuvers cannot be demonstrated, the remote mentors should be able to clearly verbalize instructions to the operating surgeon [4]. The remote mentor’s physical absence in the operating room is a major limitation if there is an adverse intra-operative event, so it should be thoroughly discussed in a mandatory pre-operative consultation involving all participants, inclusive of the patient [3]. In addition, a reliable and fast internet connection should be ensured so that there is no gap in communication between the remote mentor and the operating surgeon.

The pre-operative consult should include a discussion on procedural steps, necessary equipment, and strategies to cope with adverse events. The operating surgeon must be honest about his/her limitations and should have advanced skill sets in other laparoscopic operations [5]. The operating surgeon should also be able to perform this procedure using the open approach so that the operation can be converted if necessary [4].

We do not advocate this approach to fit all circumstances. Mature judgment is needed to choose mentors and operating surgeons who work well together [4] as well as appropriate, realistic cases [3]. 

## Conclusions

When cases are carefully selected, remote mentoring may be a way to promote minimally invasive procedures in countries where human resources and expertise are limited, but careful case selection, good mentor-mentee matching, and exceptional communication are required.
